# Non-Contact Temperature Control System Applicable to Polymerase Chain Reaction on a Lab-on-a-Disc

**DOI:** 10.3390/s19112621

**Published:** 2019-06-09

**Authors:** Junguk Ko, Jae-Chern Yoo

**Affiliations:** College of Information and Communication Engineering, Sungkyunkwan University, Suwon, Gyeonggi-Do 440-746, Korea; junguk.ko@skku.edu

**Keywords:** lab-on-a-disc, polymerase chain reaction, non-contact temperature control, non-contact thermal cycling, point-of-care diagnostic applications

## Abstract

Polymerase chain reaction (PCR) and the visual inspection of fluorescent amplicons for detection are commonly used procedures in nucleic acid tests. However, it has been extremely challenging to incorporate PCR onto a lab-on-a-disc (PCR–LOD) as it involves controlling the complicated and precise heating steps during thermal cycling and the measurement of reagent temperature. Additionally, a non-contact temperature control system without any connecting attachments needs to be implemented to facilitate the rotation of the PCR–LOD. This study presents a non-contact temperature control system to integrate conventional PCR onto an LOD. The experimental results demonstrate that our proposed system provides one-stop detection capabilities for *Salmonella* with a stable PCR amplification in a single PCR–LOD.

## 1. Introduction

The concept of a lab-on-a-disc (LOD) has received a lot of attention as a suitable and feasible methodology to fulfill the needs of point-of-care testing (POCT) as it can harness centrifugal forces through a simple rotation. Notably, centrifugation is a separation process that is required in most molecular diagnoses. For example, DNA purification after cell lysis requires reagents to be separated into the supernatant, which contains the DNA, and the debris [1]. LOD has remarkable capabilities, such as the propulsion, mixing, and aliquoting of fluids once a centrifugal force is applied [2,3,4]. One of the most prominent problems of incorporating the polymerase chain reaction (PCR) technique on an LOD is that it requires a non-contact temperature control system to enable the rotation of the LOD. Most LODs are made of polymeric compounds, such as polydimethylsiloxane or polycarbonate, with a thickness of more than 0.6 mm. These materials possess low thermal conductivity, which prevents a precise temperature measurement of the reagents inside the LOD from outside the interface. Because of its low thermal conductivity, PCR is difficult to integrate on an LOD, even though it is a specific and sensitive DNA amplification method that is widely used for diagnoses. In the past decade, several attempts have been made to integrate PCR on an LOD with outstanding results. Emmanuel Roy et al. [5] developed an integrated lab-on-a-disc with the PCR amplification by using Peltier elements for heating and cooling the reagents in the disc. This method required a vacuum to connect the Peltier elements to the surface of the disc and also wired thermocouples to measure the temperature of the reagents. To create a vacuum for the LOD system, additional instruments, such as an air pump, might be required. This makes a larger system that consumes more power to operate. Furthermore, the wired thermocouples disturb the rotation of the disc. Guanghui Wang et al. [6] developed PCR-based centrifugal microfluidics using bidirectional flow control. A resistance heating element and a thermistor were used for the contact temperature control system. However, implanting the heating element and the thermistor inside the disc may cause an imbalance state during the rotation of the centrifugal microfluidics. Even though an infrared thermometer is an appropriate non-contact instrument that can be applied to control the heating system on an LOD, it has low accuracy when measuring a lustrous surface, which causes a diffused reflection of infrared radiation [7]. The disadvantages of integrating a PCR on an LOD include not only the lack of accurate temperature control, but also the presence of PCR inhibitors. Attaching a thin metallic film around the heating chamber may overcome the low thermal conductivity of the LOD, but the PCR amplification is inhibited by certain metallic ions, such as copper and aluminum, despite their high thermal conductivity [8]. Thus, the PCR amplification chamber should consist of anti-inhibitors with a high thermal conductivity. Despite these shortcomings of incorporating the PCR on an LOD—i.e., the complex thermal control procedure and PCR inhibitors—it is still a practical method for detecting pathogens because its primers can be easily designed. PCR requires only two simple primers, namely forward and reverse primers. In contrast, the loop-mediated isothermal amplification (LAMP), which is a type of isothermal DNA amplification, requires six primers for a single target. Moreover, PCR even enables the amplification of unknown targets, i.e., when there is no information about the DNA sequence of a target genome via random amplification of polymorphic DNA (RAPD) [9]. Hence, PCR assures the amplification of non-specific targets, which further guarantees the developmental potential of the practical applications of POCT.

In this study, we proposed a non-contact temperature control system that realizes accurate thermal cycles for an end-point polymerase chain reaction on a lab-on-a-disc (PCR–LOD) to detect *Salmonella*, which includes processes such as cell lysis, purification, PCR amplification via a non-contact controlled thermal condition, and detection in order to directly obtain verifiable results. The proposed system used an infrared thermometer to determine the moment at which the reagents inside the PCR–LOD reach the required temperature for PCR amplification. A laser module was applied to heat the reagents with a graphite sheet, which efficiently absorbed the heat emitted from the laser module. Finally, we verified our work by comparing the results of *Salmonella* DNA amplification (obtained with an amplicon fluorescence) against the results obtained using a conventional tube-based PCR method.

## 2. Materials and Methods

### 2.1. Reagents and Samples

We cultured *Salmonella typhi* (OD_600_ = 0.8; 10^8^ CFU/mL) at 37 °C in a tryptone soya broth (Oxoid, Basingstoke, United Kingdom). The cultured *Salmonella* in tryptone soya broth was made into 1 mL aliquots. The precipitate of *Salmonella* was extracted by centrifugation at 13,000 rpm for 1 min, then the Simplex Easy DNA kit (Gen-IAL GmbH, Troisdorf, Germany), which is a reagent for cell lysis, was added as a preparation for experiments on PCR–LOD. We followed the protocols from the Simplex Easy DNA kit and the PCR premix kit (iNtRON Biotechnology, Seongnam, Korea) for DNA amplification as per their manufacturer’s instructions. The forward primer was 5′-GTTGAGGATGTTATTCGCAAAGG-3′ and the reverse primer was 5′-GGGTCAAGGCTGAGGAAGGT-3′ (Thermo Fisher, Waltham, MA, USA). PCR was performed at a total volume of 28 μL, in which the target gene (*Salmonella typhimurium* KCCM 11862: the size of the amplicon is about 65 bp) was amplified using the PCR premix kit. The reaction solution composed of 10 μL of the PCR reaction mixture, 10 μL of distilled water, 1 μL of the *Salmonella* DNA template, 2 μL of the primer set, and 5 μL of EvaGreen 20× in water (Biotium, Fremont, CA, USA).

### 2.2. Design and Fabrication of the Microfluidic Disc

**Figure 1:**
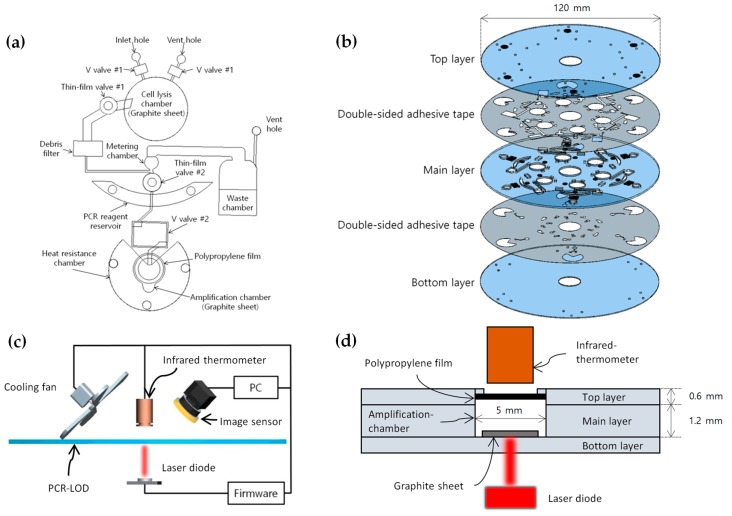
(**a**) The schematic design of a single polymerase chain reaction (PCR) amplification module of the lab-on-a-disc (PCR–LOD). (**b**) The assembly view of the PCR–LOD contained six PCR amplification modules. (**c**) The experimental setup of the non-contact temperature control system for the PCR–LOD where a heating source and an IR sensor were fixed and positioned by rotating the PCR–LOD with a step motor. (**d**) A detailed schematic illustration of the non-contact heating system.

The PCR–LOD was designed using a computer-aided design software (Pro/ENGINEER). It was composed of one 1.2 mm thick polycarbonate disc (main layer) and two 0.6 mm thick polycarbonate discs (top and bottom layers), all of which were bonded with double-sided adhesive tape (Tesa, Norderstedt, Germany) trimmed by a laser-cutting machine (Gravograph, Rillieux-la-Pape, France). We inserted V-shaped ethylene vinyl acetate valves (V valves), which were closed using a laser module to seal the reagents in each chamber, onto the main layer. Thin-film valves, made from thin vinyl and torn when the laser is applied [1], were used as opening valves to allow the fluids to flow to the next phase. We attached a 100 μm thick polypropylene film spray coated with a black coating on the top of the amplification chamber with UV-curable adhesive. Black coatings are well known for having the highest emissivity for IR measurement, which is ~1 [10]. The micro-thinned polypropylene film with the black coating guarantees reliable heat transfer from the chamber to the outside and thus makes the temperature measurement possible by an infrared thermometer with high fidelity even from the outside. Notably, the high temperature of the reagents in the amplification chamber diminished the bonding power of the double-sided adhesive tape. To protect the adhesive tape from thermal damage, the heat resistance chamber around the amplification chamber was filled with 20 μL of UV-curable adhesive, which exhibited a higher heat resistance than the adhesive tape. UV-curable adhesive remained in the 0.1 mm thick heat resistance chamber, whose capillary effect was much higher than the 1.2 mm thick amplification chamber. These modifications allowed the PCR–LOD to maintain its layers without any separation. The entire system consisted of six layers (as shown in Figure 1b), which were aligned and merged to form a single PCR–LOD. Our system consumed at most about 6 watts of power when the laser was turned on.

### 2.3. Temperature Control System

A non-contact temperature control system that consists of a heating source and a thermometer is specifically required to rotate the PCR–LOD immediately without preparation. We therefore used an infrared thermometer (Melexis, Ieper, Belgium) and a laser module to fabricate a non-contact thermal control system for the PCR amplification. The laser module generated a laser beam towards the graphite sheet to heat the reagents inside the amplification chamber efficiently. The system kept the laser module active until the temperature of the reagents in the amplification chamber was appropriate for the necessary denaturation, annealing, and extension processes to occur.

**Figure 2:**
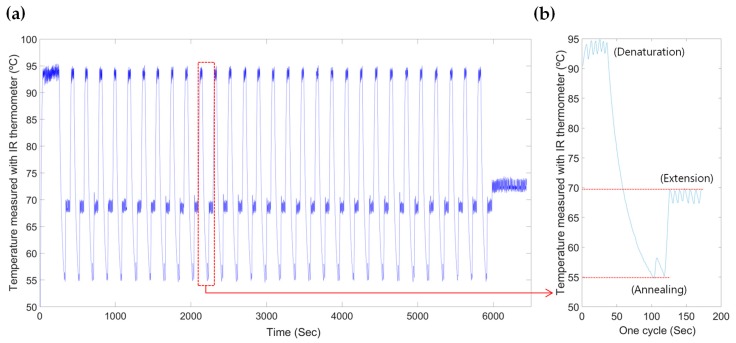
(**a**) Thermal cycles of the reagents on the proposed PCR–LOD during the PCR amplification. (**b**) A detailed view of a thermal cycle that includes denaturation, annealing, and extension temperatures.

Initial denaturation was carried out at the beginning stage of the PCR amplification to sufficiently separate the double-strand DNA into a single-strand DNA for 120 s. After we amplified the DNA template for 30 thermal cycles, the rest of the unshaped DNA strand was synthesized into the double-strand as the form of the original DNA template during the final extension stage as depicted on Table 1 and Figure 2. The infrared thermometer, whose field of view was 10° for measuring the local temperature, faced the black-painted polypropylene film over the amplification chamber (as shown in Figure 1d) and relayed the thermal information back to the firmware, which controlled the thermal cycles during the amplification process by operating a laser module. A cooling fan was activated to decrease the temperature and shorten the amplification time. This device reduced the temperature of the reagents in the amplification chamber locally.

### 2.4. Microfluidic LOD Operation

We alternated between clockwise (CW) and counterclockwise (CCW) rotations to propel fluids accurately based on the Coriolis effect. The process of cell lysis, purification, DNA amplification, and detection using the PCR–LOD is shown in Table 2. The mechanical system consisted of a motor (Swiss Amiet, Seoul, Korea), a laser module, firmware to control the laser module, and an infrared thermometer to measure the temperature of the black-painted polypropylene film on the amplification chamber. We implemented a detection procedure that examined the illumination of a fluorophore excited by a blue LED (presented as step 12 in Table 2). The entire process took approximately 110 min.

### 2.5. Optical Detection System

We adopted a fluorescent dye-based PCR using EvaGreen, which originally has an orange color, and used it to visually and qualitatively analyze the amplification results. The negative product emits an orange color, whereas the positive one exhibits a green fluorescence. This method is more effective in detecting *Salmonella* than the electrophoresis because of its convenience since it eliminates the need to extract amplicons from PCR–LOD, which may cause cross-contamination, and shortens the verification time of the results. We analyzed the RGB color variations of the amplicons using a commercial image sensor (Novomax, Shanghai, China) with an amber filter. By analyzing the RGB colors of the amplicons stimulated by a blue LED, we were able to determine whether the specimen contained *Salmonella* DNA.

## 3. Results and Discussion

### 3.1. Sealing of Reagents

**Figure 3:**
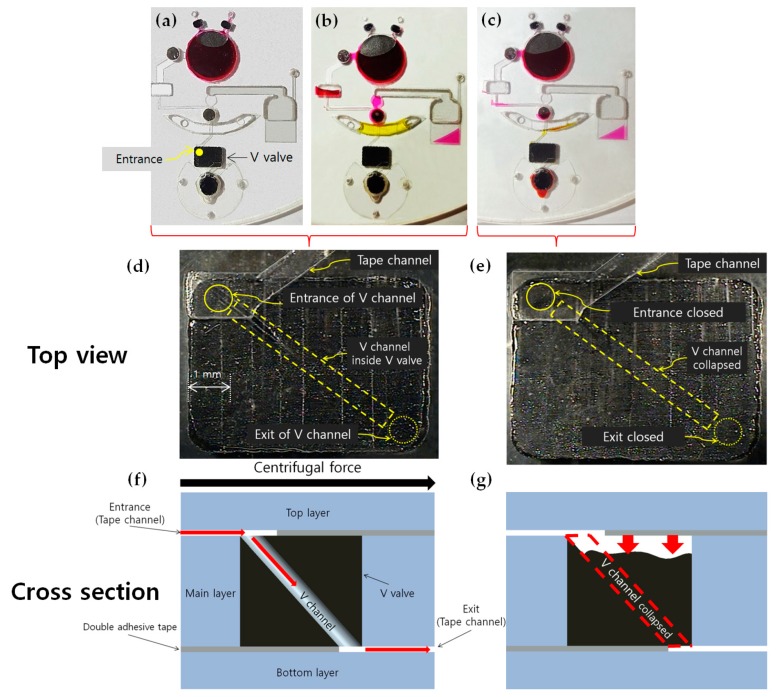
The schematic illustration of the flow of fluids in the PCR–LOD during the PCR amplification process: (**a**) The cultured *Salmonella* (dissolved in Simplex reagent (red)) was loaded, V valve no. 1 was closed, and the PCR–LOD was spun to purify the heated cell lysis reagents; (**b**) the thin-film valve no. 1 was opened and the PCR–LOD was spun so that the supernatant with purified DNA could fill the metering chamber. The PCR premix (yellow) was loaded in the PCR reagent reservoir and the thin-film valve no. 2 was then opened; (**c**) the PCR–LOD was spun so that the mixed reagents (purified DNA and PCR premix) flowed into the amplification chamber, then the V valve no. 2 was closed. Enlarged top view of the V valve; (**d**) reagents can flow from the entrance to the exit through the V channel; (**e**) the V valve closed by the laser module; (**f**) cross-sectional illustration corresponding to (**d**); (**g**) cross-sectional illustration corresponding to (**e**).

As the temperature increases, the reagents tend to be evaporated and pressurized rapidly because of thermal expansion. Therefore, the sealing of reagents is a crucial pre-process that needs to be carried out without any fluid leakage for accurate temperature control. Thus, we used ethylene vinyl acetate (EVA), which is a kind of thermoplastic, as the main material for the V valve. EVA is a suitable thermoplastic material for the PCR–LOD because it exhibits a low melting point and adheres strongly to the surface of the PCR–LOD, which makes the valve seal tightly. In addition, the V valve can be closed simply through the radiation emitted from the same laser module used for heating. In this study, we excavated a tunnel, defined as the V channel, that diagonally passed through the EVA using a 0.2 mm diameter microneedle (Figure 3d,f). We observed that the diagonally long structure easily collapsed the V channel and then reliably and tightly sealed the chambers (Figure 3e,g). Therefore, the laser module melted the V valve to block the whole path of the V channel designed in the diagonal direction. The V valve continuously withstood the pressure of the thermal expansion, heating at 97 °C for 5 min in the endurance test.

### 3.2. Cell Lysis and DNA Purification

**Figure 4:**
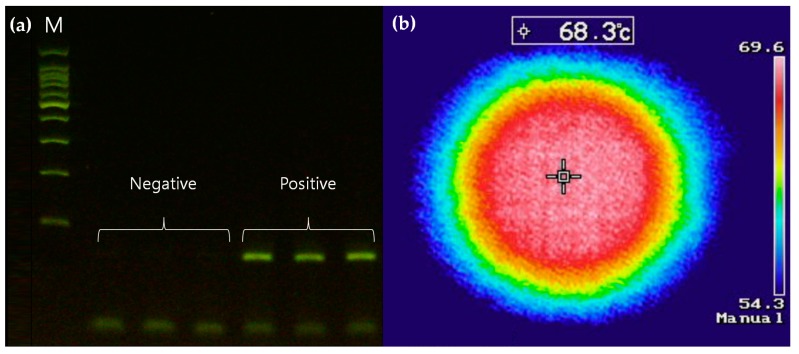
(**a**) The electrophoresis results of the PCR amplification using a thermocycler after the lysis reaches 65 °C for 3 min in a microcentrifuge tube. A negative result represents a lysis without *Salmonella*, whereas a positive result contains *Salmonella*. (**b**) The temperature of the cell lysis chamber measured by an infrared camera (Keysight, Santa Rosa, CA, USA) during the heating process with a laser module, which tells when the temperature in the cell lysis chamber reaches above 65 °C.

**Figure 5:**
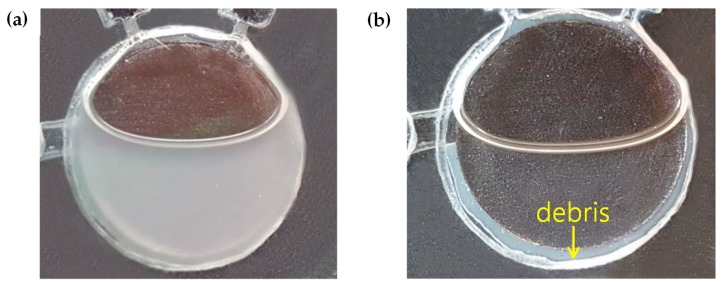
The purification process of the *Salmonella* DNA template after cell lysis: (**a**) A highly turbid solution of *Salmonella* before purification, (**b**) separation of the supernatant containing purified DNA from cell debris after LOD rotation.

Successful PCR amplifications rely on elaborate cell lysis and purification processes to obtain a pure target DNA. Here, cell lysis was achieved using the Simplex Easy DNA kit, which dissolved the *Salmonella* precipitate from the culture medium, and purification was achieved via centrifugation, which extracted the purified DNA. After the *Salmonella* culture medium was centrifuged, the precipitate was dissolved in the reagent, and then the mixture was heated with a laser module to fractionate the cells. The enzyme in the reagent is activated at temperatures above 65 °C, as shown in Figure 4a. Since the temperature reached around 68 °C during heating by the laser module in the cell lysis chamber (Figure 4b), a thermometer was not installed in the chamber. Purification was carried out by rotating the disc after cell lysis, which separated the supernatant and the cell debris effectively (Figure 5). We used a spectrophotometer (Thermo Fisher, Waltham, MA, USA) to measure the DNA yield after cell lysis and purification processes. It typically requires a minimum volume of 200 μL of DNA solution for an adequate measurement. The supernatant was collected in a tube to make a total volume of 200 μL. The amount of DNA was approximately 22.3 μg and it, in turn, corresponded to the DNA concentration of about 110 ng/μL. The debris filter, which was deeper than the connected channel, eliminated the residual debris of the supernatant in the cell lysis chamber during the fluid flow. The purified DNA template was metered at approximately 1 μL for the PCR amplification.

### 3.3. Temperature Control System for Thermal Cycling

A vast majority of PCR methods rely on thermal cycling, which activates specific enzymes at certain temperature ranges. Here, we were able to determine the temperature of PCR reagents in the amplification chamber by measuring the temperature of the black-painted polypropylene film with an infrared thermometer and setting up a laser module during the amplification heating process. The infrared thermometer continuously received thermal information from the polypropylene film, which was much thinner and thus had a higher thermal conductivity than the 0.6 mm thick disc that was exposed through the partially eliminated cavity on the top disc layer. We selected a polypropylene film instead of metallic films, because the latter aggravate the PCR amplification by interfering with the DNA synthesis. Thus, a polypropylene film was used as the lid for the amplification chamber to monitor the temperature of the reagents. Unlike metallic films, polypropylene films are widely used to bioengineer products for PCR processes and thus do not interfere with the amplification process.

### 3.4. Optical Detection

**Figure 6:**
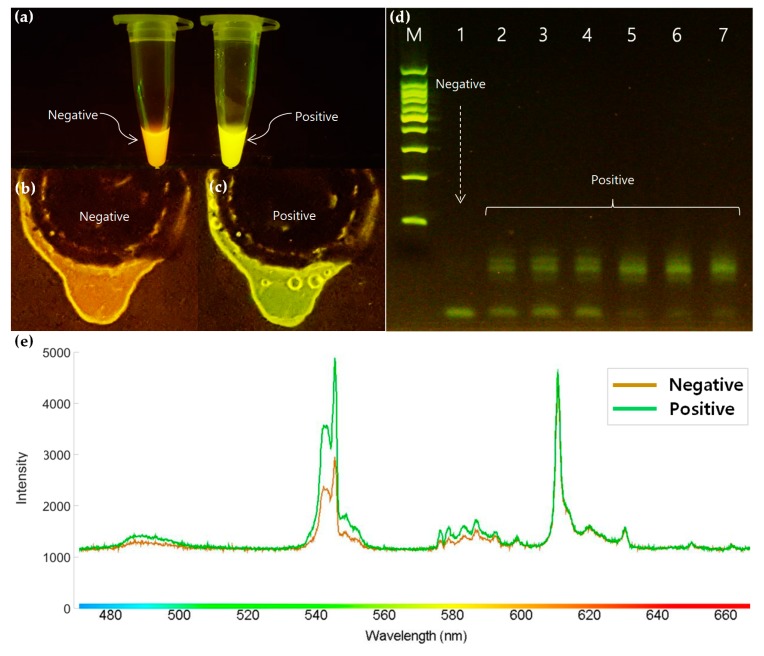
Fluorescence variations under blue LED and an amber filter: (**a**) Tube-based results of negative and positive PCR amplifications, (**b**) the PCR product after negative amplification on a PCR–LOD, (**c**) the PCR product after positive amplification on a PCR–LOD, (**d**) comparison of the electrophoresis results with positive and negative on where (M) represents 100 bp marker, (**e**) spectral properties between the negative and positive amplicons of PCR–LOD.

The amplicon extraction from PCR–LODs for detection purposes is a counterintuitive process as it exposes the amplicons to the external environment, which can cause cross-contamination under field diagnostic conditions. Moreover, the purpose of this system is to simplify laborious operations and automate the entire process until distinguishable results are achieved. With our proposed system, positive results radiate fluorescence under blue LED light after the PCR amplification. As shown in Figure 6e, the intensity of the wavelength between 540 and 550 nm and the range of the fluorescent color sharply increased after positive amplification. The fluorophore of EvaGreen radiates fluorescence when the PCR amplification is successfully conducted. Therefore, the increased intensity of fluorescence confirmed that the PCR amplification was successfully implemented. This fluorescence can also be clearly observed with the naked eye after being subjected to an amber filter, as shown in Figure 6c. Thus, the PCR–LOD can perform an automated *Salmonella* detection on a disc platform after the PCR amplification.

## 4. Conclusions

We developed a PCR–LOD device to detect *Salmonella*, in which we could control the thermal conditions without additional attachments. This study emphasized the fact that major nucleic acid amplification processes, such as cell lysis, purification, amplification, and optical detection, can be carried out on a single PCR–LOD. The optical detection of the amplicon fluorescence eliminated the need for a labor-intensive gel electrophoresis process. In addition, the entire process was executed automatically without exposing the specimens, thus protecting the samples from the external environment and ensuring reliable experimental results. Therefore, this system is easy to operate even with limited expertise. Furthermore, the V valve was designed to withstand high-pressure conditions induced from thermal expansion during the heating process. Hence, the PCR–LOD enables a stable and reliable lysis and amplification with no fluid leakage. Our system’s limit of detection is relatively worse than in gel electrophoresis. This is because our detection system simply depends on color change in the EVA green dye, which allows us to detect with the naked-eye, but it is advantageous for its portability, simplicity, and cost effectiveness. Our future research will seek to improve the proposed approaches in areas such as quantifying the minimum number of cycles to detect fluorescence, enhancing the detection limitations, and finally reducing the PCR amplification time. Nevertheless, we believe that our current proposed non-contact temperature control system for PCR amplification will contribute to the facilitation of portable microfluidic systems.

## Figures and Tables

**Table 1:** Thermal cycle temperatures for PCR amplification on PCR–LOD.

Step	PCR Phase	Temperature (°C)	Time (s)	Cycle
1	Initial denaturation	94	120	1
2	Denaturation	94	20	30
3	Annealing	55	10	30
4	Extension	69	30	30
5	Final extension	72	300	1

**Table 2:** The experimental procedure for the *Salmonella*-detecting microfluidic platform.

Step	Procedure	Spin Speed (rpm)	Time (s)
1	Load the cultured *Salmonella*, which was dissolved in the Simplex reagent for cell lysis via the inlet hole	-	-
2	Close V valve no. 1 with a laser module to seal the lysis chamber	-	10
3	Heat the lysis chamber with a laser module to accelerate the lysis reaction	-	180
4	Spin the disc counterclockwise (CCW) (the reagent is separated into the supernatant containing the purified DNA)	10,000	60
5	Open the thin-film valve no. 1	-	10
6	Spin the disc clockwise (CW) (the supernatant flows through the debris filter and reaches the metering chamber)	3000	10
7	Load the PCR premix into the PCR reagent reservoir	-	-
8	Open the thin-film valve no. 2	-	10
9	Spin the disc CW (the PCR reagents, now blended with the purified DNA template, flow through the V valve no. 2 and reach the amplification chamber)	3000	10
10	Close the V valve no. 2 with a laser module to seal the amplification chamber	-	10
11	Heat the amplification chamber according to the thermal cycles	-	6400
12	Observe the fluorescence of the amplicon excited using a blue LED	-	-
